# Antibiotic‐producing plant‐associated bacteria, anti‐virulence therapy and microbiome engineering: Integrated approaches in sustainable agriculture

**DOI:** 10.1111/1751-7915.70025

**Published:** 2024-10-09

**Authors:** Amalia Roca, Laura Monge‐Olivares, Miguel A. Matilla

**Affiliations:** ^1^ Facultad de Farmacia, Department of Microbiology Campus Universitario de Cartuja, Universidad de Granada Granada Spain; ^2^ Institute of Biotechnology, Biomedical Research Center (CIBM) University of Granada Granada Spain; ^3^ Department of Biotechnology and Environmental Protection Estación Experimental del Zaidín, Consejo Superior de Investigaciones Científicas Granada Spain

## Abstract

Plant health is crucial for maintaining the well‐being of humans, animals and the environment. Plant pathogens pose significant challenges to agricultural production, global food security and ecosystem biodiversity. This problem is exacerbated by the impact of climate change, which is expected to alter the emergence and evolution of plant pathogens and their interaction with their plant hosts. Traditional approaches to managing phytopathogens involved the use of chemical pesticides, but alternative strategies are needed to address their ongoing decline in performance as well as their negative impact on the environment and public health. Here, we highlight the advancement and effectiveness of biocontrol strategies based on the use of antimicrobial‐producing plant‐associated bacteria, anti‐virulence therapy (e.g. quorum quenching) and microbiome engineering as sustainable biotechnological approaches to promote plant health and foster sustainable agriculture. Notably, Enterobacterales are emerging as important biocontrol agents and as a source of new antimicrobials for potential agricultural use. We analysed here the genomes of over 250 plant‐associated enterobacteria to examine their potential to synthesize secondary metabolites. Exploration of the plant microbiome is of major interest in the search for eco‐friendly alternatives for reducing the use of chemical pesticides.

## Graphical abstract

Antimicrobial‐producing plant‐associated bacteria, anti‐virulence therapy and microbiome engineering as sustainable biotechnological approaches to promote plant health and foster sustainable agriculture.

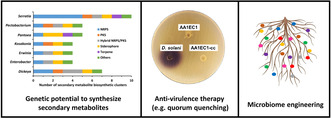

## THE NEED FOR SUSTAINABLE MANAGEMENT STRATEGIES AGAINST PLANT PATHOGENS IN THE CONTEXT OF CLIMATE CHANGE

Plants constitute more than 80% of the human diet and serve as the main feed source for livestock, but they are also a major source of pharmaceutical drugs and are crucial for sustaining healthy ecosystems (Rizzo et al., 2021). As such, maintaining plant health is an essential component of the One Health strategy to guarantee optimal health for humans, animals and the environment (Compant et al., 2024; Centers for Disease Control and Prevention [CDC], 2024). However, agricultural production systems currently face significant challenges. Among them, plant pathogenic bacteria, fungi, viruses and nematodes cause losses of up to 40% in major crops worldwide (Savary et al., 2019), resulting in estimated annual losses surpassing USD220 billion (Singh, Delgado‐Baquerizo, et al., 2023). Therefore, the incidence of these plant diseases represents a major problem for primary production and global food security, but also in terms of biodiversity of natural ecosystems (Singh, Delgado‐Baquerizo, et al., 2023). Additionally, with current predictions indicating a requirement for an agricultural production increase of up to 70% in the forthcoming years to meet the food demands of a growing global population (Hunter et al., 2017; van Dijk et al., 2021), plant pathogens pose a significant hurdle not only to scale up agricultural output, but also to sustaining current production levels. This problem is further exacerbated by the consequences of climate change, which is expected to affect the evolution and emergence of new plant pathogens and vectors, promote the spread of phytopathogens into new regions and have important consequences on the modification of plant–pathogen interactions, for example, by altering the biochemistry, physiology and molecular signalling of pathogens and plant hosts (Chaloner et al., 2021; Desaint et al., 2021; Singh, Delgado‐Baquerizo, et al., 2023).

Given these impending challenges, effective plant disease managements are crucial to guarantee an optimal crop production. Traditional methods of management involve the use of chemical pesticides, cultivating crop varieties resistant to diseases, practising intercropping and crop rotation, among other strategies. However, the decrease in the efficiency of these approaches, as well as the negative health and environmental impacts of agrochemical use (Ahmed et al., 2024; Singh, Delgado‐Baquerizo, et al., 2023), makes it necessary to implement alternative and/or complementary strategies for the control of plant diseases. In this regard, the plant microbiome is key to plant growth and health (Compant et al., 2024; Jian et al., 2024; Li et al., 2023; Mendes et al., 2013). In particular, the rhizosphere microbiome is considered as the first line of defence against soil‐borne phytopathogens (Bakker et al., 2020). Accordingly, suppressive soils protect plants against diseases as a result of the composition and activities of their associated microbiota (Bakker et al., 2020; Banerjee & van der Heijden, 2023). The mechanisms by which beneficial plant‐associated microbes carry out this protective activity are diverse and include the activation of plant immunity, production of bioactive secondary metabolites, volatile compounds and lytic enzymes, or competing for space and nutrients (Ahmed et al., 2024; Bernal, 2024; Compant et al., 2024; Jian et al., 2024; Rangel & Leveau, 2024; Singh, Delgado‐Baquerizo, et al., 2023; Zenteno‐Alegría et al., 2024; Zhang et al., 2023). Given the extensive biocontrol potential of plant‐associated microbes, there is a rising interest in their use as a strategy to reduce the use of chemical pesticides (Bravo & Soberón, 2023; Compant et al., 2024; Roca & Matilla, 2023; Zenteno‐Alegría et al., 2024).

In this opinion article, we analyse the potential of different biotechnological approaches based on the use of beneficial plant‐associated microorganisms to advance the development of strategies for promoting plant health and sustainable agricultural approaches.

## THE PLANT MICROBIOME AS A SOURCE OF NOVEL ANTIMICROBIAL COMPOUNDS

Actinobacteria currently represent the main source of antimicrobials of clinical, agricultural and industrial interest (De Simeis & Serra, 2021; van der Meij et al., 2017). Indeed, a number of Actinobacteria‐based biopesticides are commercially available (Compant et al., 2024; Kaari et al., 2023; Rey & Dumas, 2017). Nonetheless, machine learning approaches have been recently developed to mine microbial genomes and metagenomes, predicting thousands of novel antimicrobial peptides (AMPs) (Ma et al., 2022; Santos‐Júnior et al., 2024). These identified AMPs were found to be highly habitat‐specific, with soil and plant‐associated bacteria being a major source of AMPs (Santos‐Júnior et al., 2024). Similar machine learning‐based approaches have also been used to identify AMPs active against plant pathogenic bacteria (Shao et al., 2024). In addition, genomics‐based analyses on tens of thousands of genomes and metagenome‐assembled genomes uncovered that only ~3% of all bacterial secondary metabolite production has been experimentally explored (Gavriilidou et al., 2022). Among some of the most prolific regarding their potential in secondary metabolite biosynthesis were bacteria belonging to the *Pseudomonas, Burkholderia* and *Bacillus* genera (Gavriilidou et al., 2022), which are widely recognized for their biocontrol potential in agriculture (Ahmed et al., 2024; Bakker et al., 2020; Compant et al., 2024; Elshafie & Camele, 2021; Keshmirshekan et al., 2024; Oni et al., 2022; Wang, Luo, et al., 2023; Zhang et al., 2023). For instance, a recent research identified the ability of a *Pseudomonas mosselii* isolate from the rice rhizosphere to protect rice plants from bacterial (*Xanthomonas oryzae*) and fungal (*Magnaporthe oryzae*) phytopathogens, in both greenhouse and field trials (Yang et al., 2023). This biocontrol capacity resulted from *P. mosselii*'s capacity to produce the pyrazolotriazine‐type antimicrobial, pseudoiodinine. Through elucidating the regulation of pseudoiodinine synthesis, an engineered *P. mosselii* variant was generated to increase pyrazolotriazine production levels by over 22‐fold compared to the parental strain. Field trials applying purified pseudoiodinine to rice plants underscored its efficacy against fungal and bacterial phytopathogens, highlighting its promising future as a biopesticide (Yang et al., 2023). Another study revealed the extensive potential of strains of *Burkholderia ambifaria* to produce different bioactive secondary metabolites, including numerous compounds with antimicrobial properties. Among them, cepacin A production by *B. ambifaria* was shown to protect pea plants against the phytopathogenic oomycete *Pythium ultimum* (Mullins et al., 2019). Similar to other bacterial biocontrol genera like *Pseudomonas* and *Bacillus*, certain *Burkholderia* species can be pathogenic to animals, humans and plants (Elshafie & Camele, 2021). Efforts have been directed towards attenuate their virulence (Mullins et al., 2019), and developing genetically modified strains capable of heterologous expression of *Burkholderia* biosynthetic gene clusters has been undertaken to facilitate their biotechnological utilization (Petrova et al., 2022).

Plant environments are highly complex and dynamic, and subject to a diverse array of stresses, environmental cues and fluctuations in nutrient levels and composition. As a result, the plant microbiota is highly specialized and adapted to thrive within this ecological niche (Bai et al., 2015; Compant et al., 2024; Rico‐Jiménez et al., 2024; Wen et al., 2023). Moreover, there is a high complexity of interactions, both cooperative and competitive, between microbes inhabiting plants (Compant et al., 2024; Mesny et al., 2023; Poppeliers et al., 2023; Rangel & Leveau, 2024; Van Goethem et al., 2024). In these interactions, different antimicrobial metabolites play a pivotal role, actively contributing to the survival and establishment of microbiota within plant hosts (Andrić et al., 2023; Getzke et al., 2023; Hansen et al., 2022; Helfrich et al., 2018). In this context, bacterial taxa such as Enterobacterales are emerging as important biocontrol agents and as a source of novel antimicrobials of potential use in sustainable agriculture. For example, the rhizobacterium *Serratia plymuthica* A153 devotes ~5% of its genome to produce secondary metabolites, which encompass compounds with antifungal, antibacterial, anti‐oomycete and nematicidal properties (Matilla et al., 2016). Moreover, the biosynthetic complexity of specific *S. plymuthica* antimicrobials has been used for the experimental design of new biologically active metabolites (Mabesoone et al., 2024). In addition, the biosynthetic gene cluster responsible for producing the broad‐spectrum antifungal herbicolin A has been identified in various strains of *Pantoea agglomerans* (Matilla et al., 2023; Xu et al., 2022). Herbicolin A‐producing *P. agglomerans* protected wheat plants from the phytopathogenic fungus *Fusarium graminearum*, and the application of the purified antifungal provided protection to tomato fruits, as well as strawberry and barley plants, against *Botrytis cinerea* and *Magnaporthe oryzae* (Xu et al., 2022). The potential of herbicolin A as a biopesticide in agriculture has prompted the study of the regulatory circuits that control its production in *P. agglomerans* (Matilla et al., 2023; Wang, Zhou, et al., 2023), leading to a more than five‐fold increase in production levels compared to the wild‐type strain (Wang, Zhou, et al., 2023). Remarkably, other genera within the Enterobacterales are also contributing to the discovery of novel antimicrobials with potential agricultural applications. For instance, a biosynthetic cluster responsible for the biosynthesis of the new antifungal solanimycin has been identified, which is widely distributed within the *Dickeya* genus (Matilla et al., 2022). Solanimycin exhibits potent activity against a diverse array of phytopathogenic fungi (Matilla et al., 2022) and possesses unprecedented synthesis chemistry (Murphy et al., 2023), offering the potential for engineering new bioactive natural products of both agricultural and clinical significance.

**FIGURE 1:**
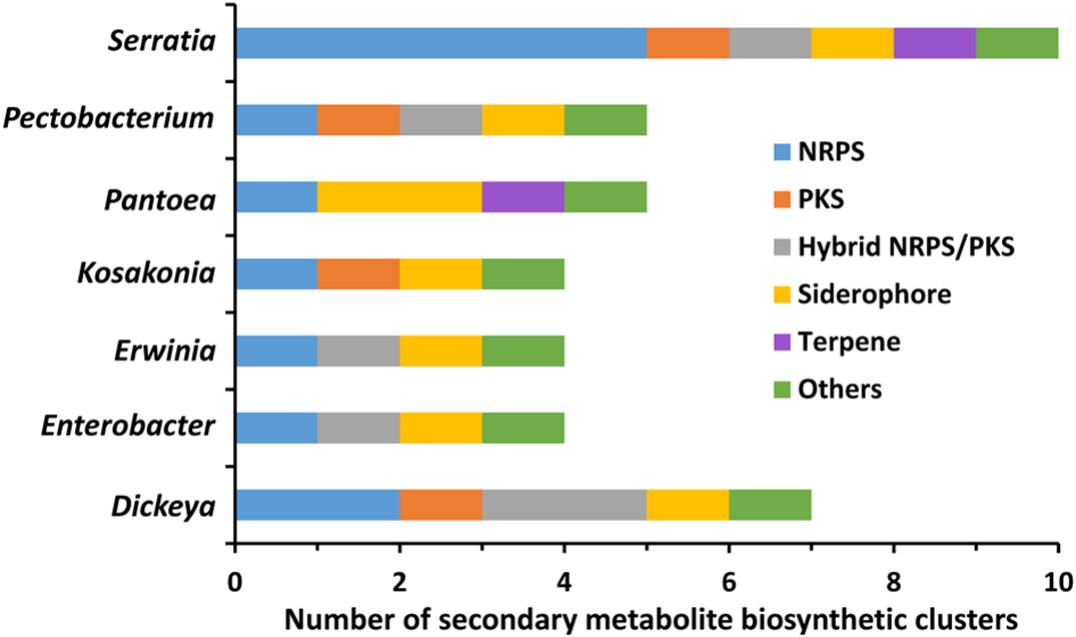
Biosynthetic diversity in plant‐associated bacteria within the order Enterobacterales. The data show the mode (excluding ‘zero’ values) of the number of different categories of biosynthetic assemblies according to the analysis with antiSMASH 7.0 (Blin et al., 2023). Top 7 producers are shown. NRPS, non‐ribosomal peptide synthetases; PKS, polyketide synthases. The category ‘Others’ includes bacteriocins, ribosomally synthesized and posttranslationally modified peptides (RiPP), β‐lactams and phenylpyrroles. Complete information is available in Table S1.

Given that the order Enterobacterales has been recognized as enriched in secondary metabolites biosynthetic clusters (Gavriilidou et al., 2022), we specifically analysed the biosynthetic potential of plant‐associated Enterobacterales by evaluating the biosynthetic capabilities of 259 plant isolates within this order, spanning 28 genera (Table S1). This analysis revealed that plant isolates belonging to the *Serratia* and *Dickeya* genera, as well as other genera such as *Pectobacterium, Pantoea* or *Erwinia*, exhibit significant potential for secondary metabolite synthesis (Figure 1). This includes the production of antimicrobials like oocydin A, andrimid, solanimycin, zeamine, prodigiosin, herbicolin A, althiomycin or pyrrolnitrin, among others (Table S1). Importantly, several biosynthetic gene clusters in the analysed plant‐associated bacteria have the potential to produce novel bioactive metabolites (Table S1). A gene cluster responsible for producing the terpene‐type volatile organic compound (VOC) sodorifen was specifically found in *S. plymuthica* (Table S1). Although the biological function of most microbial VOCs remains unknown, they have been shown to act as intra‐ and inter‐kingdom signal molecules, as well as to exhibit a diverse array of activities on various prokaryotic and eukaryotic organisms (Lammers et al., 2022; Weisskopf et al., 2021). To date, the biological function of sodorifen is unknown, but its biosynthesis in *S. plymuthica* is influenced by the presence of plant‐associated bacteria and fungi (Kai & Piechulla, 2018; Schmidt et al., 2017), suggesting a role in inter‐organismic communication. Collectively, plant‐associated Enterobacterales are a prolific source of bioactive secondary metabolites. In accordance with these data, genomic studies across 168 plant isolates of the *Pseudomonas* genus revealed their heightened potential for secondary metabolite synthesis, in contrast to non‐plant isolates (Stringlis et al., 2018). Notably, the exploration of the diversity of biosynthetic gene clusters for secondary metabolites in over 35,000 environmental metagenomes, including plant‐associated metagenomes, revealed significant habitat specificity, highlighting the ecological specialization of microbial secondary metabolism (Bagci et al., 2024). Hence, the exploration of plant microbiota is acquiring increasing interest in the search for new metabolites with biological properties of clinical and biotechnological interest (Rangel & Leveau, 2024; Zotchev, 2024).

## QUORUM QUENCHING AS AN ANTI‐VIRULENCE THERAPY APPROACH AGAINST PLANT PATHOGENS

Anti‐virulence therapy offers an alternative strategy to combat microbial pathogens compared to antimicrobial compounds. It involves targeting key microbial processes for host colonization and disease development. This targeting can be achieved, for example, by using small molecules that act on virulence determinants or by interfering with signalling cascades controlling virulence (Ellermann & Sperandio, 2020; Krell & Matilla, 2022; Muñoz‐Cazalla et al., 2023). Since anti‐virulence‐based approaches attenuate virulence without directly affecting growth, they impose less selective pressure on microbial populations for resistance development compared to antimicrobial treatments (Ellermann & Sperandio, 2020; Krell & Matilla, 2022).

**FIGURE 2:**
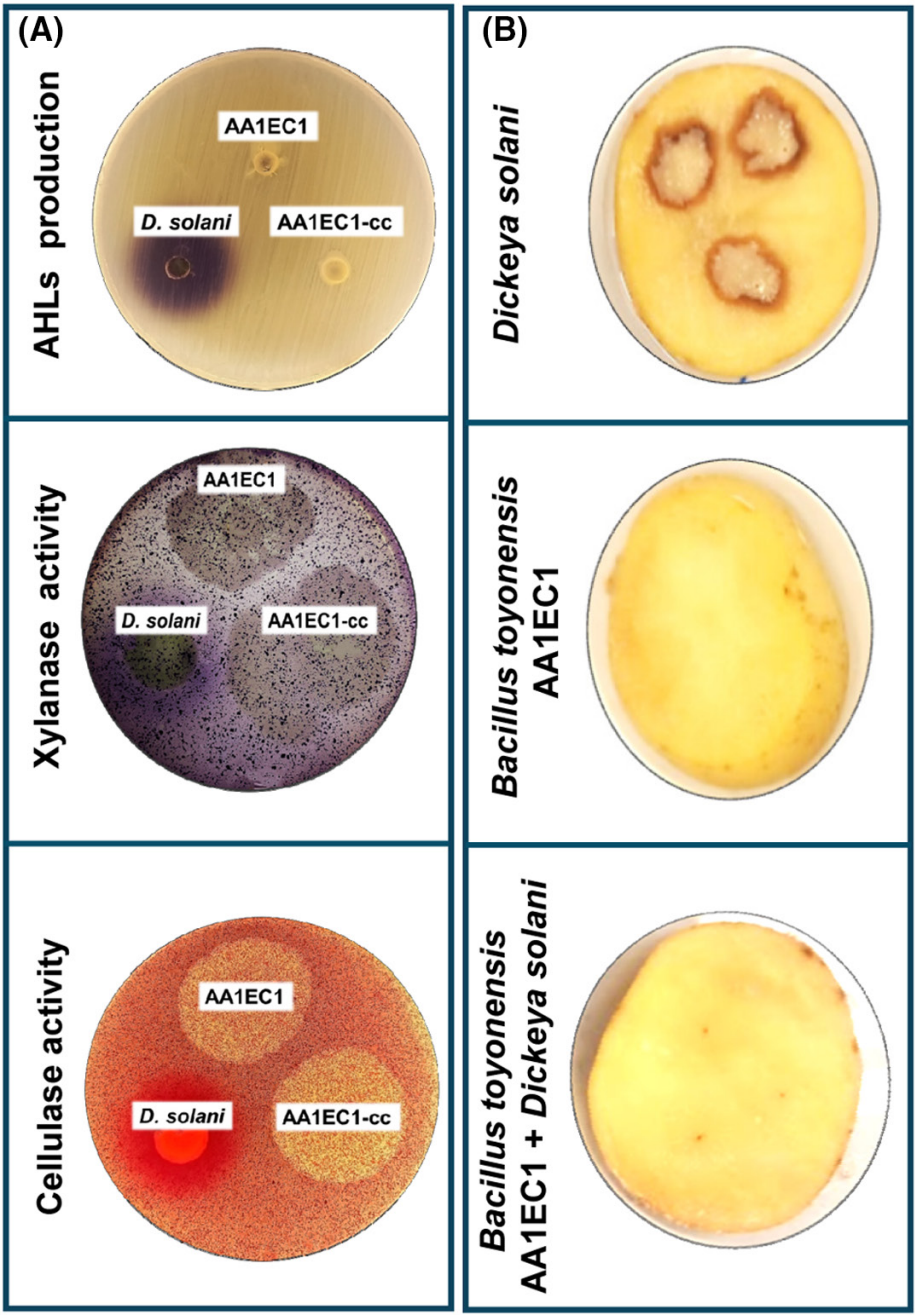
The quorum quenching properties of the plant isolate *Bacillus toyonensis* AA1EC1 protect potato tubers against the plant pathogen *Dickeya solani*. (A) Top: Detection of acyl‐homoserine lactones (AHLs) production by the biosensor strain *Chromobacterium violaceum* CV026. Middle and bottom: Xylanase and cellulase assay plates comparing *D. solani*, *B. toyonensis* AA1EC1 and a mixture of both strains. On each plate, the halos surrounding the cells are representative of AHL production (purple halo) and enzymatic activities (xylanase (purple halo) and cellulase (red halo)). (B), Impact of inoculation with *D. solani*, *B. toyonensis* AA1EC1 and a mixture of both strains on disease development in potato tubers. Photographs were taken after 2 days of infection. Full data available at Roca et al. (2024)).

Bacterial motility and chemotaxis play vital roles in the virulence of plant pathogens (Matilla & Krell, 2024) and serve as promising targets for anti‐virulence therapy strategies (Matilla & Krell, 2023). Notably, computational analyses have shown that phytopathogens possess over twice as many chemotaxis receptors compared to non‐plant‐associated bacteria (Sanchis‐López et al., 2021). Alternatively, quorum sensing (QS) is a mechanism by which bacteria coordinate their physiology and metabolism in response to population density by synthesizing and sensing specific signal molecules. QS plays an important role in the virulence of different agronomically relevant pathogens (Azimi et al., 2020; Hartmann et al., 2024; Liu et al., 2022) and also represents an attractive target in anti‐virulence strategies. Certainly, quorum quenching (QQ) is emerging as a promising approach against plant pathogens, functioning by interfering the synthesis and/or sensing of QS signals or through their inactivation (Hartmann et al., 2024; Zhang et al., 2023; Zhu et al., 2022). In this context, two recent articles published in *Microbial Biotechnology* have examined the QQ abilities of various bacterial biocontrol agents by targeting different types of QS systems found in agriculturally significant phytopathogens. In a first study, the QQ capabilities of a *Bacillus toyonensis* isolate from a halophilic plant were demonstrated against a broad spectrum of synthetic acyl‐homoserine lactones (AHLs), activities that correlated with the ability of the plant endophyte to produce different QQ enzymes, namely various lactonases and an acylase (Roca et al., 2024). *B. toyonensis* also interfered the AHLs produced by important bacterial phytopathogens such as *Pectobacterium carotovorum*, *Pectobacterium atrosepticum*, *Pseudomonas syringae* and *Dickeya solani* (Figure 2A). Remarkably, this QQ activity led to a reduction or inhibition of QS‐associated traits in these phytopathogens, such as the production of indole‐3‐acetic acid and several plant cell wall degrading exoenzymes (PCWDE) (Figure 2A), and inhibition or attenuation of their virulence in potato (Figure 2B), carrot and tomato plants. In addition, the authors showed that the heterologous expression of the lactonase BtAiiA from *B. toyonensis* in *P. syringae* reduced the production of various virulence determinants and attenuated infection in tomato plants (Roca et al., 2024).

An alternative QS system to the one mediated by AHLs is the virulence factor modulating (VFM) system, found exclusively in the *Dickeya* genus (Liu et al., 2022; Nasser et al., 2013). The VFM system regulates key virulence processes like bacterial motility and the production of PCWDE and antimicrobial compounds (Liu et al., 2022; Matilla et al., 2022), rendering it a compelling target for QQ strategies. To counteract the fact that the chemical structure of the VFM signal is unknown, Liu, Hu, et al. (2023) developed a fluorescence‐based biosensor sensitive to the VFM signal. This biosensor was successful in identifying two rhizosphere bacterial isolates of the *Pseudomonas chlororaphis* and *Enterobacter asburiae* species that efficiently quenched the VFM signal. This quenching resulted in the reduction of the expression of PCWDE‐related genes and exoenzyme production in *Dickeya zeae*. Notably, both *P. chlororaphis* and *E. asburiae* rhizosphere isolates efficiently attenuated the virulence caused by *D. zeae, D. oryzae, D. dadantii* and *D. fangzhongdai* across various agriculturally relevant crops, namely rice and banana seedlings, potato and taro, respectively (Liu, Hu, et al., 2023).

Taken together, these two studies support that the combined use of biocontrol agents targeting different QS systems is a promising approach to increase the efficiency and range of pathogens of these biological treatments. It is worth noting that while targeting QS has been proposed as an alternative to antibiotics for anti‐virulence therapy, various studies highlighted the relevance of QS in bacterial competitive fitness and growth in vivo (García‐Contreras et al., 2016; LaSarre & Federle, 2013). This aspect could potentially promote resistance development (García‐Contreras et al., 2016; LaSarre & Federle, 2013), particularly in scenarios where QS influences bacterial growth and competition. This concern extends beyond QS targeting alone, as it was theorized that anti‐virulence strategies might ultimately lead to resistance selection (García‐Contreras et al., 2016).

## RHIZOSPHERE MICROBIOME ENGINEERING FOR CROP PROTECTION

The rhizosphere microbiome is a key player in providing plants with tolerance to numerous abiotic (e.g. salinity, drought, heavy metals) and biotic (e.g. attack by phytopathogens) stresses (Compant et al., 2024; Li et al., 2023; Mesny et al., 2023; Zenteno‐Alegría et al., 2024; Zhang et al., 2023). Its enormous significance is evidenced by the proposal to integrate soil microbiomes into One Health policies (Singh, Yan, et al., 2023).

Rhizosphere microbiome engineering (RME) involves altering or adjusting rhizosphere microbial communities to improve plant health, growth and crop yield. Traditionally, this approach relied on the inoculation of single microorganisms with beneficial properties. Yet, advancements in high‐throughput cultivation methods and different omics approaches have led to the evolution of RME towards the use of more complex microbial inoculants. This involves, for example, selecting microbes based on a number of complementary plant‐beneficial traits such as their capacity to alleviate environmental stresses (e.g. drought, salinity, temperature, flooding), solubilize nutrients and synthesize phytohormones and bioactive antimicrobial compounds (Berruto & Demirer, 2024; Chialva et al., 2022; Compant et al., 2024; Jansson et al., 2023; Prigigallo et al., 2023; Wang et al., 2024).

The composition and functionality of the rhizosphere microbiome is shaped by factors like host‐related traits, environmental and soil conditions, and microorganism interactions. Understanding these traits is critical for optimizing the effectiveness of RME approaches (Berruto & Demirer, 2024; Jansson et al., 2023). For example, a recent deconstruction of a synthetic community comprising 185 phylogenetically diverse bacterial members revealed the importance of an auxin‐degrading locus within the *Variovorax* genus. This locus was found to be essential for balancing bacterial‐derived auxin levels and ensuring the functionality of the synthetic community on root growth (Finkel et al., 2020). Similarly, the assembly of a synthetic community using bacterial isolates from a disease‐suppressive soil facilitated the identification of key bacterial constituents and a secondary metabolite biosynthetic gene cluster as central players in protecting host plants against fungal phytopathogens (Carrión et al., 2019). Nonetheless, one of the main problems in microbial inoculants is the lack of consistency in their efficiency under field conditions, both in terms of growth promotion and biocontrol (Berruto & Demirer, 2024; Compant et al., 2024; Jansson et al., 2023). To help predict the success of microbial inoculants in agricultural settings, a recent study employed arbuscular mycorrhizal fungi (AMF) as a model (Lutz et al., 2023). The authors analysed the chemical, physical and biological characteristics of 54 maize fields, including soil fungal microbiomes and alterations in soil microbiota composition following AMF inoculation. The study revealed that fields with low microbial carbon content and high abundance of plant pathogenic fungi experienced greater benefits from AMF inoculations (Lutz et al., 2023). Consequently, analysing key physico‐chemical and biological soil parameters may serve as a diagnostic tool to enhance the reliability of microbial inoculants under field conditions.

Upon infection, plants can recruit beneficial microbes that provide defence against phytopathogens, a strategy often referred to as the ‘cry for help’ (Liu et al., 2024; Liu, Tao, et al., 2023; Mesny et al., 2023; Zhang et al., 2023). This selective microbial recruitment frequently involves alterations in root exudation profiles (Rolfe et al., 2019; Vismans et al., 2022). Indeed, one suggested approach for RME entails the use of genetically modified plants to secrete specific compounds, thus shaping the rhizosphere microbiome (Berruto & Demirer, 2024; Jansson et al., 2023). In this context, metabolomic approaches allowed the identification of metabolites enriched in the rhizosphere of healthy plants compared to that of diseased plants. These metabolites were subsequently applied as prebiotics in soils to reduce bacterial disease occurrence in various agriculturally relevant crops. The mechanism behind the protective effect of these prebiotics stemmed from their ability to promote the growth of commensal microbes in the rhizosphere, enhance microbial diversity and enrich several microbial functional pathways crucial for survival and competition within the niche (Wen et al., 2023). Importantly, recent research delved into the effectiveness of attenuated phytopathogens in inducing the assembly of a protective rhizosphere microbiome against plant diseases (Liu et al., 2024). Exposing *Arabidopsis* plants to non‐pathogenic *Pseudomonas syringae* mutant variants resulted in important shifts in root exudation profiles. These changes in root exudate composition led to significant alterations in the rhizosphere microbiome, which were primarily characterized by an enrichment in bacteria of the *Devosia* genus, ultimately resulting in plant growth promotion and disease suppression. Remarkably, harmless secreted metabolites from *P. syringae* avirulent variants as well as the flagellin‐derived peptide flg22 effectively triggered a protective response in *Arabidopsis* plants (Liu et al., 2024). Collectively, these findings highlight the agricultural biotechnological potential of prebiotics, non‐pathogenic bacterial strains and specific innocuous bacterial determinants as agents fostering a disease‐suppressive soil microbiome.

## CONCLUDING REMARKS AND FUTURE PERSPECTIVES

Effective management of plant diseases is essential for sustaining plant productivity and guaranteeing food security. The use of biopesticides, including live microorganisms and their byproducts, is rapidly emerging as an alternative to chemical pesticides, with global sales growth rates estimated at up to 20% (Marrone, 2019). Nonetheless, one of the main problems associated with the use of biopesticides is that they continue to be highly variable on plant performance under field conditions (Compant et al., 2024; Jansson et al., 2023; Poppeliers et al., 2023). In addition, the introduction of non‐native microbes can have a negative impact on the native soil microbiome since it can negatively influence the functioning of the local ecosystem (Jansson et al., 2023). However, recent progress in different ‘omics approaches is enabling increased understanding of: (i) microbial metabolism and physiology within plant niches; (ii) interactions among microorganisms in plant holobionts, defined as the ecological unit formed by the plant and its associated microbiota; (iii) the compatibility of microorganisms with plant hosts; and (iv) the impact of soil physico‐chemical parameters on microbial functioning (Lutz et al., 2023; Mesny et al., 2023; Poppeliers et al., 2023; Spooren et al., 2024). Research progress in this area will be crucial to enhance the efficacy of biopesticides. This aspect holds significant relevance in the current context of climate change (e.g. elevated CO_2_, temperature increases, variations in relative humidity and soil moisture) as it is expected to substantially influence the structure, resilience and functioning of plant microbiota (Jansson et al., 2023; Singh, Delgado‐Baquerizo, et al., 2023).

## AUTHOR CONTRIBUTIONS

**Amalia Roca:** Funding acquisition; writing – review and editing; project administration; data curation; conceptualization; formal analysis; investigation. **Laura Monge‐Olivares:** Investigation; writing – review and editing; data curation; methodology; formal analysis. **Miguel A. Matilla:** Conceptualization; investigation; funding acquisition; writing – original draft; methodology; validation; writing – review and editing; formal analysis; project administration; data curation; supervision.

## CONFLICT OF INTEREST STATEMENT

The authors declare that there is no conflict of interest.

## Supporting information


**Table S1.** Prediction of the biosynthetic potential of secondary metabolites in 259 bacterial isolates from plants belonging to Enterobacterales order. The analysis was performed using antiSMASH 7.0 (Blin et al. (2023) *Nucleic Acids Research* 51: W46‐W50; access April 2024). The types of biosynthetic assemblies have been grouped according to the presence of biosynthetic enzymes encoded in the corresponding gene clusters (e.g. non‐ribosomal peptide synthetases (NRPS), polyketide synthases (PKS)) and independently of the possible biological activity of the metabolites produced by the corresponding biosynthetic assemblies. For simplicity, categories resulting from antiSMASH 7.0 such as thiopeptides, aryl polyenes, beta‐lactones, homoserine lactones, phosphonate, butyrolactones, opine‐like metallophores, redox cofactors were not included in this table.

## Data Availability

The data that supports the findings of this study are available in the supplementary material of this article.
