# Supplementary material for: Antibiotic‐producing plant‐associated bacteria, anti‐virulence therapy and microbiome engineering: Integrated approaches in sustainable agriculture
Source: Microb Biotechnol. 2024 Oct 9;17(10):e70025. doi: 10.1111/1751-7915.70025 (PMC11462315; doi:10.1111/1751-7915.70025)
Supplement: Supplementary file 1 — Table S1. Prediction of the biosynthetic potential of secondary metabolites in 259 bacterial isolates from plants belonging to Enterobacterales order. The analysis was performed using antiSMASH 7.0 (Blin et al. (2023) Nucleic Acids Research 51: W46‐W50; access April 2024). The types of biosynthetic assemblies have been grouped according to the presence of biosynthetic enzymes encoded in the corresponding gene clusters (e.g. non‐ribosomal peptide synthetases (NRPS), polyketide synthases (PKS)) and independently of the possible biological activity of the metabolites produced by the corresponding biosynthetic assemblies. For simplicity, categories resulting from antiSMASH 7.0 such as thiopeptides, aryl polyenes, beta‐lactones, homoserine lactones, phosphonate, butyrolactones, opine‐like metallophores, redox cofactors were not included in this table. [file MBT2-17-e70025-s001.docx]

**Supplementary Table S1: Prediction of the biosynthetic potential of secondary metabolites in 259 bacterial isolates from plants belonging to Enterobacterales order.** The analysis was performed using antiSMASH 7.0 (Blin *et al.* (2023) *Nucleic Acids Research* 51: W46-W50; access April 2024). The types of biosynthetic assemblies have been grouped according to the presence of biosynthetic enzymes encoded in the corresponding gene clusters (e.g. non-ribosomal peptide synthetase (NRPS), polyketide synthase (PKS)) and independently of the possible biological activity of the metabolites produced by the corresponding biosynthetic assemblies. For simplicity, categories resulting from antiSMASH such as thiopeptides, arylpolyenes, betalactones, homoserine lactones, phosphonate, butyrolactones, opine-like metallophores, redox cofactors were not included in this table.

| **Strain** | **Origin/**  **isolation^a^; (phyto)pathogen**^b^ | **Non-ribosomal peptide synthetases (NRPS)^c^** | **Polyketide synthases (PKS)^c^** | **Hybrid non-ribosomal peptide synthetase- polyketide synthases NRPS/**  **PKS^c^** | **Siderophores^c^** | **Terpenes^c^** | **Bacteriocins, ribosomally synthesized and post-translationally modified peptides (RiPP), β-lactam, phenylpyrroles^c^** | **Total number of clusters** | **NCBI Bioproject Accession No.** |
| --- | --- | --- | --- | --- | --- | --- | --- | --- | --- |
| ***Atlantibacter hermannii* DDE1** | Root tissue *Cucurbita pepo;* members of this species can be human pathogens | 1 (enterobactin) | - | - | - | 1 (carotenoid) | 1  (RiPP-like) | 3 | PRJNA272572 |
| ***Atlantibacter subterranea* ZYL01** | Paddy soil | 1 (frederiksenibactin  -like) | - | - | - | 1 (carotenoid) | 1  (RiPP-like) | 3 | PRJNA553324 |
| ***Buttiauxella* sp. A111** | Forest soil | 1 (frederiksenibactin  -like) | - | - | - | - | - | 1 | PRJDB8128 |
| ***Buttiauxella* sp. UBA1641** | Wood | 1 (unknown) | - | - | - | - | - | 1 | PRJNA348753 |
| ***Cedecea* sp. NFIX57** | Rizoplane | - | - | - | 1  (aerobactin) | - | 1  (RiPP-like) | 2 | PRJEB20280 |
| ***Citrobacter amalonaticus* S646** | Leafy green;  members of this species can be human pathogens | 2 (frederiksenibactin  -like & unknown) | - | - | - | - | 1  (RiPP-like) | 3 | PRJNA431620 |
| ***Citrobacter braakii* MiY-A** | Coriander; members of this species can be plant and human pathogens | 1 (enterobactin) | - | - | - | - | - | 1 | PRJNA541977 |
| ***Citrobacter freundii* LDL3-3** | Bean sprouts; members of this species can be plant and human pathogens | 1 (enterobactin) | - | - | - | - | - | 1 | PRJNA597197 |
| ***Citrobacter* sp. BDA59-3** | Rice leaves; members of this genus can be plant and human pathogens | 1 (enterobactin) | - | - | - | - | 1  (RiPP-like) | 2 | PRJNA670042 |
| ***Citrobacter* sp. H12-3-2** | Flower shop; members of this genus can be plant and human pathogens | 1 (enterobactin) | - | - | - | - | 1  (RiPP-like) | 2 | PRJNA580050 |
| ***Citrobacter* sp. LUTT5** | Rhizosphere soil and tubers of *Solanum tuberosum;* plant growth promoting bacterium | 1 (enterobactin) | - | - | - | - | - | 1 | PRJNA600388 |
| ***Citrobacter* sp. S40-1-2** | Flower shop; members of this genus can be plant and human pathogens | 1 (enterobactin) | - | - | - | - | 1  (RiPP-like) | 2 | PRJNA580050 |
| ***Cronobacter sakazakii* GP1999** | Tomato rhizosphere; members of this species can be foodborne and human pathogens | 1 (unknown) | - | - | 1  (aerobactin) | 1 (carotenoid) | - | 3 | PRJNA258403 |
| ***Cronobacter* sp. JZ38** | *Tribulus terrestres;* plant growth promoting bacterium | 1 (unknown) | - | - | - | 1 (carotenoid) | - | 2 | PRJNA345401 |
| ***Cronobacter turicensis* UBA5650** | Wood; members of this species can be foodborne and human pathogens | 1 (unknown) | - | - | 1  (aerobactin) | 1 (carotenoid) | - | 3 | PRJNA348753 |
| ***Dickeya aquatica* 174/2** | Tomato and cucumber;  phytopathogen | 4 (bicornutin A1/A2, dichrysobactin, minimycin-like & 1 unknown) | - | 1 (solanimycin) | 1  (unknown) | - | 1 (β-lactam) | 7 | PRJEB15300 |
| ***Dickeya chrysanthemi* Ech1591** | Disease in corn (*Zea mays*); phytopathogen | 2 (indigoidine & unknown) | - | - | 1  (unknown) | - | 2  (cyanobactin-like & β-lactam) | 5 | PRJNA31295 |
| ***Dickeya chrysanthemi* NCPPB 516** | *Pathenium argentatum;* phytopathogen | 2 (dichrysobactin-like & minimycin-like) | - | - | 1  (unknown) | - | 2  (cyanobactin-like & β-lactam) | 5 | PRJNA172904 |
| ***Dickeya dadantii* DSM 18020** | *Pelargonium capitatum;* phytopathogen | 2 (minimycin-like & unknown) | - | 2 (solanamycin & unknown) | 1  (unknown) | - | 2  (RIPP-like & cyanobactin-like) | 7 | PRJNA407418 |
| ***Dickeya dadantii* 3937** | Saintpaulia plants; phytopathogen | 3 (bovienimide A-like, dichrysobactin-like & minimycin-like) | - | - | 1  (unknown) | - | 1  (cyanobactin-like) | 5 | PRJNA30 |
| ***Dickeya dianthicola* ME23** | Potato tissue sample; phytopathogen | 2 (minimycin-like & unknown) | 1  (oocydin A) | - | 1  (unknown) | - | 1  (cyanobactin-like) | 5 | PRJNA485499 |
| ***Dickeya dianthicola* NCPPB 453** | *Dianthus caryophyllus;* phytopathogen | 2 (indigoidine & unknown) | 1  (oocydin A) | - | 1  (unknown) | - | 1  (cyanobactin-like) | 5 | PRJNA172901 |
| ***Dickeya dianthicola* RNS04.9** | Potato plant (*Solanum tuberosum*) with blackleg symptoms; phytopathogen | 2 (dichrysobactin-like & minimycin-like) | 1  (oocydin A) | - | 1  (unknown) | - | 1  (RIPP-like & cyanobactin-like) | 5 | PRJNA347818 |
| ***Dickeya fangzhongdai* DSM 101947** | Pear trees (*Pyrus pyrifolia*)*;* phytopathogen | 3 (minimycin & 2 unknown) | - | 2 (zeamine & solanimycin) | 1  (unknown) | - | 1  (cyanobactin-like) | 7 | PRJNA418606 |
| ***Dickeya fangzhongdai* LN1** | Pear trees (*Pyrus pyrifolia*)*;* phytopathogen | 3 (minimycin-like & 2 unknown) | - | 2  (zeamine & solanimycin) | 1  (unknown) | - | 1  (cyanobactin-like) | 7 | PRJNA484841 |
| ***Dickeya fangzhongdai* PA1** | *Phalaenopsis* sp. orchids affected by bacterial soft rot*;* phytopathogen | 3 (minimycin-like, dichrysobactin & unknown) | - | 2  (zeamine & solanimycin) | 1  (unknown) | - | 1  (cyanobactin-like) | 7 | PRJNA380527 |
| ***Dickeya fangzhongdai* QZH3** | Pear*;* phytopathogen | 3 (minimycin-like, dichrysobactin & unknown) | - | 2  (zeamine & solanimycin) | 1  (unknown) | - | 1  (cyanobactin-like) | 7 | PRJNA484843 |
| ***Dickeya paradisiaca* Ech703** | *Solanum tuberosum;* phytopathogen | 3 (dichrysobactin  & 2 unknown) | 1  (oocydin A) | - | 1  (vibrioferrin-like) | - | - | 5 | PRJNA33069 |
| ***Dickeya paradisiaca* NCPPB 2511** | *Musa paradisiaca;* phytopathogen | 3 (dichrysobactin  & 2 unknown) | 1  (oocydin A) | - | 1  (vibrioferrin-like) | - | - | 5 | PRJNA172954 |
| ***Dickeya poaceiphila* NCPPB 569** | Sugarcane (*Saccharum officinarum*)*;* phytopathogen | 3 (minimycin-like & 2 unknown) | 1 (orofanic acid) | 2 (solanimycin & unknown) | 1  (unknown) | - | - | 7 | PRJNA556543 |
| ***Dickeya solani* D s0432-1** | *Solanum tuberosum* stem*;* phytopathogen | 2 (minimycin-like & dichrysobactin) | 1  (oocydin A) | 2  (zeamine & solanimycin) | 1  (unknown) | - | 1  (cyanobactin) | 7 | PRJNA344558 |
| ***Dickeya solani* GBBC 2040** | *Solanum tuberosum*; phytopathogen | 2 (minimycin-like & unknown) | 1  (oocydin A) | 2  (zeamine & solanimycin) | 1  (unknown) | - | 1  (cyanobactin) | 7 | PRJNA173464 |
| ***Dickeya solani* IFB 0099** | *Solanum tuberosum*; phytopathogen | 2 (minimycin-like & unknown) | 1  (oocydin A) | 2  (zeamine & solanimycin) | 1  (unknown) | - | 1  (cyanobactin-like) | 7 | PRJNA272487 |
| ***Dickeya solani* IFB0167** | *Solanum tuberosum;* phytopathogen | 2 (minimycin-like & unknown) | 1  (oocydin A) | 2  (zeamine & solanimycin) | 1  (unknown) | - | 1  (cyanobactin-like) | 7 | PRJNA611911 |
| ***Dickeya solani* IFB0223** | *Solanum tuberosum* rhizosphere*;* phytopathogen | 2 (minimycin-like & unknown) | 1  (oocydin A) | 2  (zeamine & solanimycin) | 1  (unknown) | - | 1  (cyanobactin-like) | 7 | PRJNA416044 |
| ***Dickeya solani* IFB0417** | *Solanum tuberosum;* phytopathogen | 2 (minimycin-like & unknown) | 1  (oocydin A) | 2  (zeamine & unknown) | 1  (unknown) | - | 1  (cyanobactin-like) | 7 | PRJNA611911 |
| ***Dickeya solani* IPO 2222** | *Solanum tuberosum* tissue sample with blackleg*;* phytopathogen | 2 (minimycin-like & unknown) | 1  (oocydin A) | 2  (zeamine & solanimycin) | 1  (unknown) | - | 1  (cyanobactin-like) | 7 | PRJNA317288 |
| ***Dickeya solani* MK10** | *Solanum tuberosum*; phytopathogen | 2 (dichrysobactin, minimycin-like) | 1  (oocydin A) | 2  (zeamine & solanimycin) | 1  (unknown) | - | 1  (cyanobactin-like) | 7 | PRJNA172957 |
| ***Dickeya solani* RNS 08.23.3.1.A** | *Solanum tuberosum;* phytopathogen | 2 (minimycin-like, dichrysobactin-like) | 1  (oocydin A) | 2  (zeamine & solanimycin) | 1  (unknown) | - | 1  (cyanobactin-like) | 7 | PRJNA176848 |
| ***Dickeya* sp. Secpp 1600** | Radish*;* phytopathogen | 3 (minimycin-like, dichrysobactin-like & unknown) | - | 2  (zeamine & solanimycin) | 1  (unknown) | - | 1  (cyanobactin-like) | 7 | PRJNA407418 |
| ***Dickeya zeae* CE1** | *Canna edulis* tissue sample; phytopathogen | 4 (dichrysobactin-like, indogoidine & 2 unknown) | - | 1 (solanimycin) | 1  (unknown) | - | 1  (cyanobactin-like) | 7 | PRJNA483111 |
| ***Dickeya parazeae* Ech586 (formerly, *Dickeya zeae* Ech586)** | *Philodendron;* phytopathogen | 3 (indogoidine & 2 unknown) | - | 1 (solanimycin) | 1  (unknown) | - | 1  (cyanobactin-like) | 6 | PRJNA33667 |
| ***Dickeya zeae* EC1** | Diseased rice plants*;* phytopathogen | 3 (indigoidine & 2 unknown) | 1  (oocydin A) | 1  (zeamine) | 1  (unknown) | - | 1  (cyanobactin-like) | 7 | PRJNA229184 |
| ***Dickeya zeae* MS2** | Basal pseudostems of banana plants; phytopathogen | 3 (indigoidine & 2 unknown) | - | 1 (solanimycin) | 1  (unknown) | - | 1  (cyanobactin-like) | 6 | PRJNA429264 |
| ***Dickeya zeae* NCPPB 3532** | *Solanum tuberosum;* phytopathogen | 2 (indigoidine & unknown) | - | 1 (solanimycin) | 1  (unknown) | - | 1  (cyanobactin-like) | 5 | PRJNA172951 |
| ***Edaphovirga cremea* DSM 105170** | *Codonopsis clematidea*’s rhizospheric soil | 1 (minimycin-like) | 2  (unknown) | - | 1 (desferrioxamine E) | - | 1  (RiPP-like) | 5 | PRJNA478623 |
| ***Enterobacter asburiae* AEB30** | Ginger; biocontrol agent | 1 (enterobactin) | - | 1 (unknown) | 1  (aerobactin-like) | - | - | 3 | PRJNA594005 |
| ***Enterobacter asburiae* SD4L** | Rice; phytopathogen | 1 (enterobactin) | - | 1 (unknown) | 1  (aerobactin) | - | - | 3 | PRJNA670619 |
| ***Enterobacter bugandensis* IIT-BT 08** | Leaves of a local plant near the Kharagpur railway station (India); members of this species can be human pathogens as well as plant growth promoting bacteria | 1 (enterobactin) | - | - | 1  (aerobactin) | - | - | 2 | PRJNA82637 |
| ***Enterobacter cancerogenus* MiY-F** | Coriander; members of this species can be human pathogens | 1 (enterobactin) | - | - | - | - | - | 1 | PRJNA541977 |
| ***Enterobacter cloacae* CZ-1** | Paddy soil; members of this species can be plant and human pathogens as well as plant growth promoting bacteria | 1 (unknown) | - | - | 1  (aerobactin) | - | 1  (RiPP-like) | 3 | PRJNA520972 |
| ***Enterobacter cloacae* GGT036** | Forest soil; members of this species can be plant and human pathogens as well as plant growth promoting bacteria | 1 (enterobactin) | - | - | 1  (aerobactin) | - | - | 2 | PRJNA259401 |
| ***Enterobacter cloacae* subsp. *cloacae* CAPREX_E7** | Ghanaian yam; members of this species can be plant and human pathogens as well as plant growth promoting bacteria | 1 (enterobactin) | - | 1 (unknown) | 1  (aerobactin-like) | - | - | 3 | PRJNA362587 |
| ***Enterobacter hormaechei* INSali2** | Lettuce; members of this species can be plant and human pathogens as well as plant growth promoting bacteria | 1 (enterobactin) | - | - | 1  (aerobactin) | - | 1  (RiPP-like) | 3 | PRJNA311932 |
| ***Enterobacter hormaechei* subsp. *xiangfangensis* NS371** | *Oryza sativa*; members of this species can be plant and human pathogens as well as plant growth promoting bacteria | 1 (enterobactin) | - | - | 1  (aerobactin) | - | 1  (RiPP-like) | 3 | PRJNA278240 |
| ***Enterobacter kobei* ENHKU01** | Diseased pepper (*Capsicum anuum*); phytopathogen | 1 (Frederiksenibactin-like) | - | - | 1  (aerobactin) | - | - | 2 | PRJNA170442 |
| ***Enterobacter ludwigii* AA4** | *Zea mays* root | 1 (enterobactin) | - | - | 1  (aerobactin) | - | - | 2 | PRJNA357031 |
| ***Enterobacter ludwigii* EcWSU1** | Onion bulbs with symptoms of rotting; phytopathogen | 1 (enterobactin) | - | - | 1  (aerobactin) | - | - | 2 | PRJNA67983 |
| ***Enterobacter ludwigii* I42** | Rhizospheric soil of *Lycium barbarum* | 1 (enterobactin) | - | - | 1  (aerobactin) | - | - | 2 | PRJNA532824 |
| ***Enterobacter ludwigii* JP6** | *Nicotiana tabacum* rhizosphere soil | 1 (enterobactin) | - | - | 1  (aerobactin) | - | - | 2 | PRJNA532789 |
| ***Enterobacter ludwigii* JP9** | *Nicotiana tabacum* rhizosphere soil | 1 (enterobactin) | - | - | 1  (aerobactin) | - | - | 2 | PRJNA532821 |
| ***Enterobacter ludwigii* P101** | Switchgrass (*Panicum virgatum*) | 1 (enterobactin) | - | - | 1  (aerobactin) | - | - | 2 | PRJNA210654 |
| ***Enterobacter ludwigii* UW5** | Grass’ rhizosphere | 1 (enterobactin) | - | - | 1  (aerobactin) | - | - | 2 | PRJNA285312 |
| ***Enterobacter oligotrophica* CCA6** | Leaf soil | 1 (unknown) | - | - | - | - | - | 1 | PRJDB7530 |
| ***Enterobacter pneumoniae* SBP-8** | *Sorghum bicolor* rhizosphere | 1 (enterobactin) | - | - | 1  (aerobactin) | - | - | 2 | PRJNA338268 |
| ***Enterobacter roggenkampii* KQ-01** | *Morus alba* var. *atropurpurea* root; phytopathogen | 1 (enterobactin) | - | - | 1  (aerobactin) | - | - | 2 | PRJNA644454 |
| ***Enterobacter* sp. DKU_NT_01** | *Glycine max* | 1 (frederiksenibactin  -like) | - | - | 1  (aerobactin) | - | - | 2 | PRJNA384709 |
| ***Enterobacter* sp. E20** | *Oryza sativa* glyphosate polluted soil | 1 (enterobactin) | - | - | 1  (aerobactin) | - | - | 2 | PRJNA263377 |
| ***Enterobacter* sp. HK169** | Tomato roots | 1 (enterobactin) | - | - | 1  (aerobactin) | - | - | 2 | PRJNA326573 |
| ***Enterobacter* sp. JUb54 RIT712** | Rotting apples | 1 (enterobactin) | - | - | - | - | 1  (RiPP-like) | 2 | PRJNA622270 |
| ***Enterobacter* sp. NFIX09** | Switchgrass’ rhizosphere | 1 (enterobactin) | 1 (unknown) | - | - | - | 1  (RiPP-like) | 3 | PRJEB17057 |
| ***Enterobacter* sp. R4-368** | *Jatropha* *curcas* surface sterilized roots | 2 (enterobactin & unknown) | - | - | - | - | 1  (RiPP-like) | 3 | PRJNA189171 |
| ***Enterobacter* sp. SA187** | *Indigofera argentea*’s (desert plant) root nodules | 2 (frederiksenibactin  -like & unknown) | - | - | - | 1 (carotenoid) | - | 3 | PRJNA352128 |
| ***Enterobacter* sp. SES19** | Black rice shoot | 1 (enterobactin) | - | 1 (unknown) | 1  (aerobactin) | - | - | 3 | PRJNA603425 |
| ***Enterobacter* sp. 638** | Stem of *Populus trichocarpa* | 1 (enterobactin) | - | - | - | - | - | 1 | PRJNA17461 |
| ***Erwinia amylovora* ATCC 49946** | Infected apple tree*;* phytopathogen | 1 (rhizomide A/ B/C) | - | 1 (unknown) | 1  (desferrioxamine E) | - | 1 (RiPP-like) | 4 | PRJEA43757 |
| ***Erwinia amylovora* CFPB1430** | *Crataegus* sp.*;* phytopathogen | 1 (rhizomide A/ B/C) | - | 1 (unknown) | 1  (desferrioxamine E-like) | - | 1 (RiPP-like) | 4 | PRJNA46805 |
| ***Erwinia amylovora* CTBT1-1** | Pear (*Pyrus communis*)*;* phytopathogen | 1 (rhizomide A/ B/C) | - | 1 (unknown) | 1  (desferrioxamine E) | - | 1 (RiPP-like) | 4 | PRJNA397580 |
| ***Erwinia amylovora* CTBT3-1** | Pear (*Pyrus communis*)*;* phytopathogen | 1 (rhizomide A/ B/C) | - | 1 (unknown) | 1  (desferrioxamine E) | - | 1 (RiPP-like) | 4 | PRJNA397580 |
| ***Erwinia amylovora* E-2** | *Malus* sp*;* phytopathogen | 1 (rhizomide A/ B/C) | - | 1 (unknown) | 1  (desferrioxamine E) | - | 1 (RiPP-like) | 4 | PRJNA289329 |
| ***Erwinia amylovora* FB-20** | Pear (*Pyrus communis*)*;* phytopathogen | 1 (rhizomide A/ B/C) | - | 1 (unknown) | 1  (desferrioxamine E) | - | 1 (RiPP-like) | 4 | PRJNA505563 |
| ***Erwinia amylovora* FB-86** | Apple tree*;* phytopathogen | 1 (rhizomide A/ B/C) | - | 1 (unknown) | 1  (desferrioxamine E) | - | 1 (RiPP-like) | 4 | PRJNA506631 |
| ***Erwinia amylovora* FB-207** | Pear tree*;* phytopathogen | 1 (rhizomide A/ B/C) | - | 1 (unknown) | 1  (desferrioxamine E) | - | 1 (RiPP-like) | 4 | PRJNA506635 |
| ***Erwinia amylovora* FB-307** | Apple tree*;* phytopathogen | 1 (rhizomide A/ B/C) | - | 1 (unknown) | 1  (desferrioxamine E) | - | 1 (RiPP-like) | 4 | PRJNA506636 |
| ***Erwinia amylovora* LA635** | Golden Delicious apple orchards*;* phytopathogen | 1 (rhizomide A/ B/C) | - | 1 (unknown) | 1  (desferrioxamine E) | - | 1  (RiPP-like) | 4 | PRJEB4664 |
| ***Erwinia amylovora* LA636** | Golden Delicious apple orchards*;* phytopathogen | 1 (rhizomide A/ B/C) | - | 1 (unknown) | 1  (desferrioxamine E) | - | 1  (RiPP-like) | 4 | PRJEB4665 |
| ***Erwinia amylovora* MAGFLF 2** | Apple*;* phytopathogen | 1 (rhizomide A/ B/C) | - | 1 (unknown) | 1  (desferrioxamine E) | - | 1  (RiPP-like) | 4 | PRJNA397580 |
| ***Erwinia amylovora* MASHBO** | Pear (*Pyrus communis*)*;* phytopathogen | 1 (unknown) | - | 1 (unknown) | 1  (desferrioxamine E) | - | 1  (RiPP-like) | 4 | PRJNA397580 |
| ***Erwinia amylovora* RISTBO01-2** | Apple (Malus domestica)*;* phytopathogen | 1 (rhizomide A/B/C) | - | 1 (unknown) | 1  (desferrioxamine E) | - | 1  (RiPP-like) | 4 | PRJNA397580 |
| ***Erwinia amylovora* TS3128** | *Pyrus pyrifolia* var. culta*;* phytopathogen | 1 (rhizomide A/B/C) | - | 1 (unknown) | 1  (desferrioxamine E) | - | 1  (RiPP-like) | 4 | PRJNA639906 |
| ***Erwinia amylovora* TS3238** | Pear tree*;* phytopathogen | 1 (rhizomide A/B/C) | - | 1 (unknown) | 1  (desferrioxamine E) | - | 1  (RiPP-like) | 4 | PRJNA506637 |
| ***Erwinia billingiae* Eb661** | *Pyrus communis;* phytopathogen | 1  (unknown) | - | - | 1 (desferrioxamine E) | - | - | 2 | PRJEA34781 |
| ***Erwinia persicina* B64** | Rotten onion bulb (*Allium cepa* L.)*;* phytopathogen | 2 (unknown) | 1  (unknown) | - | - | - | - | 3 | PRJNA397630 |
| ***Erwinia pyrifoliae* DSM 12163** | Necrotic tissue of *Pyrus pyrifolia;* phytopathogen | 1 (rhizomide A/B/C) | - | - | 1 (desferrioxamine E) | - | 1 (RiPP-like) | 3 | PRJEA37877 |
| ***Erwinia pyrifoliae* EpK1/15** | Apple twig*;* phytopathogen | 1 (rhizomide A/B/C) | - | - | 1 (desferrioxamine E) | - | 1 (RiPP-like) | 3 | PRJNA411857 |
| ***Erwinia pyrifoliae* Ep1/96** | Necrotic tissue of *Pyrus pyrifolia;* phytopathogen | 1 (rhizomide A/B/C) | - | - | 1 (desferrioxamine E) | - | 1 (RiPP-like) | 3 | PRJEA34779 |
| ***Erwinia* sp. Ejp617** | Nashi pear*;* phytopathogen | 1 (rhizomide A/B/C) | - | - | 1 (desferrioxamine E) | - | 1  (RIPP-like) | 3 | PRJNA51141 |
| ***Erwinia tasmaniensis* Et1/99** | Apple flowers*;* phytopathogen | 1 (unknown) | - | - | 1 (desferrioxamine E) | - | - | 2 | PRJEA20585 |
| ***Erwinia tracheiphila* BuffGH** | Infected wild gourd (*Cucurbita pepo* subsp. *texana*) plant; phytopathogen | 1 (indigoidine) | - | - | - | - | - | 1 | PRJNA272881 |
| ***Erwinia tracheiphila* MDcuke** | *Cucumis sativus* (xylem)*;* phytopathogen | 1 (unknown) | - | - | - | - | - | 1 | PRJNA272881 |
| ***Escherichia albertii* MBT-EA1** | *Lollo bionda* (lettuce); members of this species can be human and animal pathogens | 1 (enterobactin) | - | - | - | - | 1  (RiPP-like) | 2 | PRJNA433818 |
| ***Escherichia coli* O145:H28 str. RM12581** | Lettuce; members of this species can be human pathogen | 1 (enterobactin) | - | - | 1  (aerobactin) | - | - | 2 | PRJNA178647 |
| ***Escherichia coli* 06-00048** | Alfalfa sprouts; members of this species can be plant, food and human pathogens | 1 (enterobactin) | - | - | - | - | - | 1 | PRJNA241122 |
| ***Escherichia coli* 08-00022** | Bagged lettuce; members of this species can be plant, food and human pathogens | 1 (enterobactin) | - | 1 (unknown) | 1  (unknown) | - | - | 3 | PRJNA241122 |
| ***Escherichia coli* 09-00049** | Lettuce; members of this species can be plant, food and human pathogens | 1 (enterobactin) | - | - | - | - | 1  (RiPP-like) | 2 | PRJNA241122 |
| ***Franconibacter pulveris* DSM 19144** | Fruit powder | 1 (unknown) | - | - | - | 1 (carotenoid) | - | 2 | PRJNA221041 |
| ***Gibbsiella quercinecans* DSM 25889** | Inner bark of *Quercus* *petraea* displaying acute oak decline; phytopathogen | 2 (unknown) | - | - | - | - | - | 2 | PRJNA500332 |
| ***Gibbsiella quercinecans* FRB97** | Necrotic lesion of AOD affected tree  *Quercus robur;* phytopathogen | 2 (unknown) | - | - | - | - | - | 2 | PRJNA308223 |
| ***Izhakiella* sp. KSNA2** | Surface-sterilized stem tissue of *Calistegia soldanella* | 1 (unknown) | - | - | - | 1  (unknown) | 1 (RiPP-like) | 3 | PRJNA542973 |
| ***Klebsiella aerogenes* NFIX39** | Switchgrass’ rhizoplane; members of this species can be human pathogens | 1 (enterobactin) | - | - | - | - | - | 1 | PRJEB17905 |
| ***Klebsiella michiganensis* AKKL-001** | *Morus alba;* phytopathogen | 1 (enterobactin) | - | 1 (unknown) | - | - | 1 (RiPP-like) | 3 | PRJNA655785 |
| ***Klebsiella michiganensis* RC10** | Rice field; members of this species can be plant and human pathogens | 1 (unknown) | - | - | 1  (aerobactin) | - | 1 (RiPP-like) | 3 | PRJNA278789 |
| ***Klebsiella* *oxytoca* strain SA2 (formerly, *Klebsiella michiganensis* SA2)** | Pioneer grass (*Psammochloa villosa)* roots*;* members of this species can be plant and human pathogens | 2 (kleboxymycin & enterobactin) | - | - | - | - | 1 (RiPP-like) | 3 | PRJNA248712 |
| ***Klebsiella oxytoca* KCTC 1686** | Plant; members of this species can be human pathogens as well as plant growth promoting bacteria | 1 (enterobactin) | - | 1 (unknown) | - | - | 1 (RiPP-like) | 3 | PRJNA65523 |
| ***Klebsiella* sp. LTGPAF-6F** | *Alhagi sparsifolia* Shap | 1 (enterobactin) | - | 1 (unknown) | - | - | 1 (RiPP-like) | 3 | PRJNA344738 |
| ***Klebsiella* sp. MPUS7** | *Solanum tuberosum* rhizosphere soil | 2 (kleboxymycin & enterobactin) | - | 1 (unknown) | - | - | 1 (RiPP-like) | 4 | PRJNA600386 |
| ***Klebsiella* sp. M5al** | Rice roots | 2 (kleboxymycin & enterobactin) | - | 1 (unknown) | - | - | 2 (2 RiPP-like) | 5 | PRJNA361010 |
| ***Klebsiella variicola* DSM 15968** | Banana root (*Musa* sp.); members of this species can be human pathogens | 1 (enterobactin) | - | - | - | - | 1  (RiPP-like) | 2 | PRJNA272370 |
| ***Klebsiella variicola* DX120E** | Sugarcane root; members of this species can be human pathogens | 1 (enterobactin) | - | - | - | - | 1  (RiPP-like) | 2 | PRJNA259590 |
| ***Klebsiella variicola* E57-7** | Maize roots; members of this species can be human pathogens | 1 (enterobactin) | - | - | - | - | 1  (RiPP-like) | 2 | PRJNA327507 |
| ***Klebsiella variicola* F10Cl** | Switchgrass (*Panicum* *virgatum* EG1101 cultivar); members of this species can be human pathogens | 1 (enterobactin) | - | - | - | - | 1  (RiPP-like) | 2 | PRJNA257883 |
| ***Klebsiella variicola* GN02** | *Cenchrus macrourus*; members of this species can be human pathogens | 1 (enterobactin) | - | - | - | - | 2  (2 RiPP-like) | 3 | PRJNA481410 |
| ***Klebsiella variicola* 342** | Interior of nitrogen-efficient maize plants (*Zea mays*); members of this species can be human pathogens | 1 (enterobactin) | - | - | - | - | 1  (RiPP-like) | 2 | PRJNA28471 |
| ***Kluyvera genomosp* 3. PO2S7** | *Oryza sativa;* members of this species can be human pathogens | 1 (unknown) | - | - | - | - | - | 1 | PRJNA604081 |
| ***Kluyvera intermedia* HR2** | Flower; members of this species can be human pathogens | 1 (enterobactin) | - | - | - | - | 1  (RiPP-like) | 2 | PRJNA580036 |
| ***Kluyvera intermedia* N2-1** | Flower shop; members of this species can be human pathogens | 1 (enterobactin) | - | - | - | - | 1  (RiPP-like) | 2 | PRJNA580050 |
| ***Kosakonia oryzae* Ola 51** | *Oryza* *latifolia* roots | 1 (enterobactin) | - | - | 1  (xanthoferrin-like) | - | 1  (RiPP-like) | 3 | PRJNA309028 |
| ***Kosakonia pseudosacchari* BDA62-3** | Rice leaves | 2 (enterobactin & unknown) | - | - | - | - | 1  (RIPP-like) | 3 | PRJNA670042 |
| ***Kosakonia radicincitans* DSM 16656** | Winter wheat phyllosphere; members of this species can be human pathogens | 1 (enterobactin) | 1  (unknown) | - |  | - | 1  (RiPP-like) | 3 | PRJNA161109 |
| ***Kosakonia radicincitans* D4** | Rice roots; members of this species can be human pathogens | 1 (unknown) | - | - | 1  (xanthoferrin-like) | - | 1  (RiPP-like) | 3 | PRJEB19719 |
| ***Kosakonia radicincitans* GXGL-4A** | Maize rhizosphere; members of this species can be human pathogens | 1 (enterobactin) | 1  (xanthoferrin-like) | - |  | - | 1  (RiPP-like) | 3 | PRJNA316511 |
| ***Kosakonia radicincitans* YD4** | *Ilex paraguariensis* St. Hil. rhizosphere; members of this species can be human pathogens | 1 (enterobactin) | 1  (unknown) | - |  | - | 1  (RiPP-like) | 3 | PRJNA260749 |
| ***Kosakonia sacchari* BO-1** | Sweet potato | 2 (enterobactin& unknown) | - | - | - | - | 1  (RiPP-like) | 3 | PRJNA327605 |
| ***Kosakonia sacchari* DSM 107661** | Rhizosphere soil (topsoil) from rice fields | 2 (bovienimide A  & enterobactin) | - | - | - | - | 1  (RiPP-like) | 3 | PRJNA544607 |
| ***Kosakonia sacchari* SP1** | Sugarcane stem | 2 (enterobactin & unknown) | - | - | - | - | 1  (RiPP-like) | 3 | PRJNA175988 |
| ***Kosakonia* sp. MUSA4** | Banana tree leaf | 1 (unknown) | 1  (unknown) | - | - | - | 1  (RiPP-like) | 3 | PRJNA428293 |
| ***Leclercia* *adecarboxylata* LK24** | Plant | 1 (enterobactin) | - | - | 1  (aerobactin) | - | - | 2 | PRJNA277417 |
| ***Lelliottia nimipressuralis* CCUG 25894** | Elm tree (*Ulmus* sp.) | 1 (enterobactin) | - | - | - | - | 1  (RiPP-like) | 2 | PRJNA305687 |
| ***Lelliottia* sp. F153** | *Solanum tuberosum* | 1 (enterobactin) | - | - | - | - | 1  (RiPP-like) | 2 | PRJNA421661 |
| ***Mangrovibacter phragmitis* MP23** | *Phragmites karka* root tissue | 1 (enterobactin-like) | - | - | - | - | 1  (RiPP-like) | 2 | PRJNA323358 |
| ***Mangrovibacter plantisponsor* DSM 19579** | *Ponteresia coarctata* (plant related to wild rice) from mangrove ecosystem | 1 (unknown) | - | - | - | - | 1  (RiPP-like) | 2 | PRJNA456025 |
| ***Pantoea agglomerans* ASB05** | Cherry | 1 (frederiksenibactin-like) | - | - | - | - | - | 1 | PRJNA594723 |
| ***Pantoea agglomerans* CFSAN047153** | Leaf from “Rome” apple cultivar | 1 (dichrysobactin-like) | - | - | - | - | - | 1 | PRJNA243331 |
| ***Pantoea agglomerans* CFSAN047154** | Leaf from “Rome” apple cultivar | 1 (dichrysobactin-like) | - | - | - | - | - | 1 | PRJNA243331 |
| ***Pantoea agglomerans* C410P1** | Lettuce | 1 (dichrysobactin-like) | - | - | - | - | - | 1 | PRJNA335437 |
| ***Pantoea agglomerans* 9Rz4** | Oilseed rape rhizosphere | 1 (herbicolinA & unknown) | - | - | 1 (desferrioxamine E) | 1 (carotenoid) | - | 3 | PRJNA861005 |
| ***Pantoea agglomerans* L15** | *Hypericum perphoratum* phyllosphere | 1 (dichrysobactin-like) | - | - | - | - | - | 1 | PRJNA386631 |
| ***Pantoea agglomerans* 3** | *Triticum aestivum* seed | 1 (unknown) | - | - | 1 (desferrioxamine E) | 1 (carotenoid) | - | 3 | PRJNA315986 |
| ***Pantoea agglomerans* 4** | Wheat seed | 2 (frederiksenibactin-like + unknown) | - | - | 1 (desferrioxamine E) | 1 (carotenoid) | - | 4 | PRJNA255804 |
| ***Pantoea alhagi* LTYR-11Z** | *Alhagi sparsifolia* shap leaf | - | - | - | 1 (desferrioxamine E) | 1 (carotenoid) | - | 2 | PRJNA374633 |
| ***Pantoea ananatis* CFH 7-1** | Diseased greenhouse cotton boll; phytopathogen | 1 (unknown) | - | - | 2 (desferrioxamine E & aerobactin) | 2 (carotenoids) | - | 5 | PRJNA287251 |
| ***Pantoea ananatis* DZ-12** | Maize (*Zea mays*) brown rot leaves; phytopathogen | - | - | - | 2 (desferrioxamine E & aerobactin) | 2 (carotenoids) | - | 4 | PRJNA506327 |
| ***Pantoea ananatis* LMG 20103** | Blight and dieback of Eucalyptus; phytopathogen | - | - | - | 2 (desferrioxamine E & aerobactin) | 1 (carotenoid) | - | 3 | PRJNA43085 |
| ***Pantoea ananatis* NN08200** | Sugarcane stem | - | - | - | 2 (desferrioxamine E & aerobactin) | - | - | 2 | PRJNA514184 |
| ***Pantoea ananatis* PA13** | Diseased rice grain; phytopathogen | - | - | - | 2 (desferrioxamine E & aerobactin) | - | - | 2 | PRJNA74285 |
| ***Pantoea ananatis* PNA 97-1R** | Tissue sample of  *Allium cepa;* phytopathogen | - | - | - | 2 (desferrioxamine E & aerobactin) | - | - | 2 | PRJNA384061 |
| ***Pantoea ananatis* R100** | Rice seeds | - | - | - | 2 (desferrioxamine E & aerobactin) | - | 1  (RiPP-like) | 3 | PRJNA310041 |
| ***Pantoea ananatis* YJ76** | Rice | - | - | - | 2 (desferrioxamine E & aerobactin) | - | 1  (RiPP-like) | 3 | PRJNA382605 |
| ***Pantoea cypripedii* LMG 2657** | *Cypripedium* sp. (orchid) | 2  (frederiksenibactin-like & unknown) | - | - | - | 1 (carotenoid) | - | 3 | PRJNA252995 |
| ***Pantoea cypripedii* NE1** | *Sesbania* spp. root nodule | 1  (frederiksenibactin-like) | - | - | - | - | - | 1 | PRJNA417504 |
| ***Pantoea dispersa* C34** | Plant | 1  (frederiksenibactin-like) | - | - | - | 1 (carotenoid) | - | 2 | PRJNA510304 |
| ***Pantoea eucalypti* LMG 24197** | Eucalyptus leaves showing symptoms of bacterial blight and die-back in Uruguay; phytopathogen | - | - | - | 1 (desferrioxamine E) | 1 (carotenoid) | - | 2 | PRJNA503936 |
| ***Pantoea* sp. Bo_7** | *Gossypium hirsutum* leaves | - | - | - | 1 (desferrioxamine E) | 1 (carotenoid) | 1 (RiPP-like) | 3 | PRJNA563888 |
| ***Pantoea* sp. CCBC3-3-1** | Branch of *Cotinus coggygria* | 3 (unknown) | - | - | - | 1 (carotenoid) | - | 4 | PRJNA506501 |
| ***Pantoea* *phytobeneficialis* MSR2** | *Mimosa scabrella* roots | 1 (frederiksenibactin-like) | - | - | - | - | - | 1 | PRJNA416796 |
| ***Pantoea* sp. OXWO6B1** | *Avena sativa* seeds | 1 (unknown) | - | - | 2 (desferrioxamine E & aerobactin) | 1 (carotenoid) | - | 4 | PRJNA312098 |
| ***Pantoea* sp. Sc1** | Diseased cotton seed and lint tissue; phytopathogen | 1 (frederiksenibactin-like) | - | - | 1 (desferrioxamine E) | 1 (carotenoid) | - | 3 | PRJNA86999 |
| ***Pantoea stewartii* ZJ-FGZX1** | *Dracaena sanderiana* leaf; phytopathogen | 1 (unknown) | - | - | 2 (desferrioxamine E & aerobactin) | - | - | 3 | PRJNA607905 |
| ***Pantoea stewartii* subsp. *stewartii* DC283** | *Zea mays;* phytopathogen | - | - | - | 2  (aerobactin & desferrioxamine E) | 1 (carotenoid) | - | 3 | PRJNA342501 |
| ***Pantoea vagans* C9-1** | *Malus domestica*; commercially registered for biological control of fire blight | 1 (frederiksenibactin-like) | - | - | - | - | - | 1 | PRJNA43531 |
| ***Pantoea vagans* FBS135** | *Pinus massoniana* | - | - | - | - | - | - | 0 | PRJNA383128 |
| ***Pantoea vagans* LMG 24199** | Eucalyptus | 1 (unknown) | - | - | - | - | - | 1 | PRJNA505269 |
| ***Pantoea vagans* TYU1** | *Taxus cuspidata* seeds | 1 (enterobactin) | - | - | 1 (desferrioxamine E) | 1 (carotenoid) | - | 3 | PRJNA396982 |
| ***Pectobacterium actinidiae* KKH3** | Kiwi fruit*;* phytopathogen | 2  (fontizine A & unknown) | 1 (oronofacic acid) | 2 (unknown) | 1  (unknown) | - | - | 6 | PRJNA252053 |
| ***Pectobacterium atrosepticum* CFBP 6276** | *Solanum tuberosum* var. *felsina;* phytopathogen | 4  (fontizine A, amonabactin P 750, bovienimide A & 1 unknown) | 1 (oronofacic acid) | - | 1  (unknown) | - | - | 6 | PRJNA188399 |
| ***Pectobacterium atrosepticum* JG10-08** | Black leg symptoms of *Solanum tuberosum;* phytopathogen | 3  (fontizine A, amonabactin P 750 & unknown) | 1 (oronofacic acid) | - | 1  (unknown) | - | 1  (RiPP-like) | 6 | PRJNA247436 |
| ***Pectobacterium atrosepticum* SCRI1043** | Potato stem with blackleg disease symptoms; phytopathogen | 3  (fontizine A, amonabactin P 750 & unknown) | 1 (oronofacic acid) | - | 1  (unknown) | - | 1  (RiPP-like) | 6 | PRJNA350 |
| ***Pectobacterium atrosepticum* 21A** | Potato stems (*Solanum tuberosum*)*;* phytopathogen | 3  (fontizine A, amonabactin P 750 & unknown) | 1 (oronofacic acid) | - | 1  (unknown) | - | 1  (RiPP-like) | 6 | PRJNA252906 |
| ***Pectobacterium atrosepticum* 36A** | *Solanum tuberosum;* phytopathogen | 3  (fontizine A, amonabactin P 750 & unknown) | 1 (oronofacic acid) | - | 1  (unknown) | - | - | 5 | PRJNA419060 |
| ***Pectobacterium brasiliense* BC1** | Chinese cabbage*;* phytopathogen | 1 (amonabactin P 750) | - | 1  (unknown) | 1  (unknown) | - | 1  (RIPP-like) | 4 | PRJNA263220 |
| ***Pectobacterium brasiliense* BZA12** | Cucumber*;* phytopathogen | 4 (kleboxymycin, amonabactin P 750 & 2 unknown) | - | - | 1  (unknown) | - | 1  (RiPP-like) | 6 | PRJNA416681 |
| ***Pectobacterium brasiliense* HNP201719** | Potato*;* phytopathogen | 2 (amonabactin P 750 & unknown) | - | - | 1  (unknown) | - | - | 3 | PRJNA591391 |
| ***Pectobacterium brasiliense* SX309** | Cucumber*;* phytopathogen | 2 (amonabactin P 750 & unknown) | - | - | 1  (unknown) | - | - | 3 | PRJNA379343 |
| ***Pectobacterium brasiliense* 1692** | Potato*;* phytopathogen | 3 (amonabactin P 750, jessenipeptin & 1 unknown) | - | 1  (unknown) | 1  (unknown) | - | 2  (RiPP-like & β-lactam) | 7 | PRJNA599934 |
| ***Pectobacterium carotovorum* WPP14** | Potato*;* phytopathogen | 2 (amonabactin P 750 & unknown) | - | 1 (unknown) | 1  (unknown) | - | - | 4 | PRJNA615876 |
| ***Pectobacterium carotovorum* subsp. *carotovorum* BP201601.1** | Potato*;* phytopathogen | 2 (amonabactin P 750 & unknown) | - | 1  (unknown) | 1  (unknown) | - | - | 4 | PRJNA506867 |
| ***Pectobacterium carotovorum* subsp. *carotovorum* B5** | *Brassica rapa* ssp. *pekinensis* (Chinese cabbage) infected leaves*;* phytopathogen | 4 (bovienimide A, amonabactin P 750 & 2 unknown) | - | 1  (unknown) | 1  (unknown) | - | - | 6 | PRJNA269237 |
| ***Pectobacterium carotovorum* subsp. *carotovorum* JR1.1** | Radish*;* phytopathogen | 2 (amonabactin P 750 & unknown) | - | 1  (unknown) | 1  (unknown) | - | 1  (RiPP-like) | 5 | PRJNA506868 |
| ***Pectobacterium carotovorum* subsp. *carotovorum* PC1** | Chinese cabbage and other vegetables*;* phytopathogen | 1 (amonabactin P 750) | - | 1  (unknown) | 1  (unknown) | - | - | 3 | PRJNA31289 |
| ***Pectobacterium carotovorum* subsp. *carotovorum* PCC21** | Chinese cabbage*;* phytopathogen | 1 (amonabactin P 750) | - | 1  (unknown) | 1  (unknown) | - | 1  (RiPP-like) | 4 | PRJNA171960 |
| ***Pectobacterium carotovorum* subsp. *carotovorum* 67** | Deep rotting corm of *Amorphophallus konjac;* phytopathogen | 3 (fontizine A, amonabactin P 750 & unknown) | - | - | 1  (unknown) | - | - | 4 | PRJNA504724 |
| ***Pectobacterium odoriferum* BC S7** | Infected leaf of *Brassica rapa* ssp. *pekinensis* (Chinese cabbage)*;* phytopathogen | 2 (amonabactin P 750 & unknown) | - | 2 (xenocoumacin-like & unknown) | 1  (unknown) | - | - | 5 | PRJNA263297 |
| ***Pectobacterium odoriferum* JK2.1** | Kimchi cabbage*;* phytopathogen | 2 (amonabactin P 750 & unknown) | - | - | 1  (unknown) | - | - | 3 | PRJNA513207 |
| ***Pectobacterium parmentieri* HC** | Potato stem*;* phytopathogen | 1 (dichrysobactin-like) | - | - | 1  (aerobactin) | - | 1  (RiPP-like) | 3 | PRJNA591387 |
| ***Pectobacterium parmentieri* IFB5408** | *Solanum tuberosum* stem*;* phytopathogen | 1 (dichrysobactin-like) | - | - | 1  (aerobactin) | - | - | 2 | PRJNA433470 |
| ***Pectobacterium parmentieri* IFB5485** | *Solanum tuberosum;* phytopathogen | 1 (dichrysobactin-like) | - | - | 1  (aerobactin) | - | - | 2 | PRJNA433470 |
| ***Pectobacterium parmentieri* IFB5605** | *Solanum tuberosum* stem*;* phytopathogen | 1 (dichrysobactin-like) | - | - | 1  (aerobactin) | - | - | 2 | PRJNA433470 |
| ***Pectobacterium parmentieri* IFB5623** | *Solanum tuberosum* stem*;* phytopathogen | 1 (dichrysobactin-like) | - | - | 1  (aerobactin) | - | - | 2 | PRJNA433470 |
| ***Pectobacterium parmentieri* SCC3193** | *Solanum tuberosum;* phytopathogen | 1 (dichrysobactin-like) | - | - | 1  (aerobactin) | - | - | 2 | PRJNA122637 |
| ***Pectobacterium polaris* NIBIO1006** | *Solanum tuberosum* tissue sample*;* phytopathogen | 1 (amonabactin P 750) | - | 1  (unknown) | 1  (peramine-like) | - | - | 3 | PRJNA344960 |
| ***Pectobacterium polaris* NIBIO1392** | *Solanum tuberosum* tissue sample*;* phytopathogen | 1 (amonabactin P 750) | - | 1 (unknown) | 1  (unknown) | - | - | 3 | PRJNA345001 |
| ***Pectobacterium polaris* PZ1** | Potato*;* phytopathogen | 2 (amonabactin P 750 & unknown) | - | 1 (unknown) | - | - | 3  (RiPP-like, phenazine, β-lactam) | 6 | PRJNA591396 |
| ***Pectobacterium punjabense* SS95** | *Solanum tuberosum* plant stem with blackleg symptoms*;* phytopathogen | 1 (amonabactin P 750) | - | - | 1  (unknown) | - | - | 2 | PRJNA527839 |
| ***Pectobacterium versatile* SCC1** | *Solanum tuberosum* tuber*;* phytopathogen | 1 (amonabactin P 750) | - | - | 1  (unknown) | - | - | 2 | PRJNA379819 |
| ***Pectobacterium versatile* 14A** | *Solanum tuberosum* tuber*;* phytopathogen | 1 (amonabactin P 750) | - | - | 1  (unknown) | - | - | 2 | PRJNA507761 |
| ***Pectobacterium versatile* 3-2** | Rotten *Solanum tuberosum* tuber*;* phytopathogen | 2 (amonabactin P 750 & unknown) | - | - | 1  (unknown) | - | - | 3 | PRJNA264967 |
| ***Pectobacterium wasabiae* CFBP 3304** | *Eutrema japonicum* root*;* phytopathogen | 1 (unknown) | - | - | - | - | - | 1 | PRJNA320083 |
| ***Pectobacterium zantedeschiae* 2M** | *Calla lily* bulbs*;* phytopathogen | 2  (amonabactin P 750-like) | - | 2  (unknown) | - | - | 1  (RiPP-like) | 5 | PRJNA416435 |
| ***Phytobacter diazotrophicus* UAEU22** | Rhizosphere of date palm | 1 (enterobactin) | - | - | - | - | 1  (RiPP-like) | 2 | PRJNA625126 |
| ***Pseudocitrobacte*r sp. RIT 415** | Sugarcane | 1 (enterobactin) | - | - | - | - | 2  (RiPP-like) | 3 | PRJNA449743 |
| ***Raoultella ornithinolytica* DSM 16926** | *Sorghum bicolor*; members of this species can be human pathogens | 1 (enterobactin) | - | 1 (unknown) | - | - | 1  (RiPP-like) | 3 | PRJNA643785 |
| ***Raoultella terrigena* R1Gly** | Tobacco roots | 2 (enterobactin & unknown) | - | - | - | - | 1  (RiPP-like) | 3 | PRJNA254925 |
| ***Rosenbergiella nectarea* 8N4** | *Amydalus communis* (almond) floral nectar | 1 (enterobactin) | - | - | 1 (desferrioxamine E) | 1 (carotenoid) | 1  (RiPP-like) | 4 | PRJEB17013 |
| ***Salmonella enterica* subsp. *enterica* serovar *agona* str. 460004 2-1** | Unsweetened puffed-rice cereal; members of this species can be plant and human pathogens | 1 (unknown) | - | - | - | - | - | 1 | PRJNA78433 |
| ***Salmonella enterica* subsp. *enterica* serovar *anatum* str. ATCC BAA-1592** | Tomato; members of this species can be plant and human pathogens | 1 (enterobactin) | - | - | - | - | - | 1 | PRJNA62791 |
| ***Salmonella enterica* subsp. *enterica* serovar *bredeney* str. CFSAN001080** | Marjoram; members of this species can be plant and human pathogens | 1 (enterobactin) | - | - | - | - | - | 1 | PRJNA167391 |
| ***Salmonella* serovar *cubana* CFSAN002050** | Fresh alfalfa sprouts; members of this species can be plant and human pathogens | 1 (unknown) | - | - | - | - | - | 1 | PRJNA184141 |
| ***Salmonella enterica* subsp. *enterica* serovar *Thompson* str. RM6836** | Lettuce; members of this species can be plant and human pathogens | 1 (enterobactin) | - | - | - | - | - | 1 | PRJNA70669 |
| ***Serratia ficaria* NCTC12148** | Calimyrna fig | 3 (kolossin & 2 unknown) | - | 1  (unknown) | 1  (unknown) | - | 1  (RiPP-like) | 6 | PRJEB6403 |
| ***Serratia fonticola* CPSE11** | Root of *Codonopsis pilosula* | 3 (frederiksenibactin-like & 2 unknown) | 1 (unknown) | - | 1  (aerobactin) | - | - | 5 | PRJNA611944 |
| ***Serratia fonticola* GS2** | Sesame rhizosphere | 3 (frederiksenibactin-like & 2 unknown) | - | - | 1  (aerobactin) | - | - | 4 | PRJNA308245 |
| ***Serratia fonticola* UPMP2124** | Spinach | 3 (unknown) | - | - | 1  (aerobactin) | - | - | 4 | PRJNA642017 |
| ***Serratia grimesii* BXF1** | Pinewood nematode, Bursaphelenchus xylophilus; plant growth promoting bacterium | 2 (frederiksenibactin-like & unknown) | 1  (unknown) | - | 1  (unknown) | - | 2  (RiPP-like & pyrrolnitrin) | 6 | PRJEB21173 |
| ***Serratia liquefaciens* FG3** | Flower of *Stachytarpheta glabra* | 5 (frederiksenibactin-like & 4 unknown) | - | - | 1  (unknown) | - | - | 6 | PRJNA505252 |
| ***Serratia liquefaciens* S1** | Mixed greens | 3 (frederiksenibactin-like & 2 unknown) | - | - | 1  (unknown) | - | - | 4 | PRJNA559804 |
| ***Serratia marcescens* BP2** | *Jatropha curcas* seeds; members of this species can be plant and human pathogens as well as plant growth promoting bacteria | 4 (unknown) | - | 1  (prodigiosin) | - | - | - | 5 | PRJNA612043 |
| ***Serratia marcescens* B3R3** | *Zea mays*; members of this species can be plant and human pathogens as well as plant growth promoting bacteria | 5 (unknown) | - | 1  (prodigiosin) | - | - | - | 6 | PRJNA299742 |
| ***Serratia marcescens* CAPREX_SY21** | Ghanaian yam; members of this species can be plant and human pathogens as well as plant growth promoting bacteria | 5 (unknown) | - | 1  (prodigiosin) | - | - | - | 6 | PRJNA374552 |
| ***Serratia marcescens* JW-CZ2** | Rhizosphere soil of tea tree; members of this species can be plant and human pathogens as well as plant growth promoting bacteria | 5 (unknown) | - | 1  (prodigiosin) | - | - | - | 6 | PRJNA638538 |
| ***Serratia marcescens* RSC-14** | Surface-sterilized roots of *Solanum nigrum*; members of this species can be plant and human pathogens as well as plant growth promoting bacteria | 4 (kolossin & 3 unknown) | - | 2  (prodigiosin & unknown) | - | - | - | 6 | PRJNA294721 |
| ***Serratia marcescens* SOLR4** | Solanacea rhizosphere; members of this species can be plant and human pathogens as well as plant growth promoting bacteria | 5 (kolossin & 4 unknown) | - | 1  (prodigiosin) | - | - | - | 6 | PRJNA428295 |
| ***Serratia marcescens* S11** | Cucumber; members of this species can be plant and human pathogens as well as plant growth promoting bacteria | 5 (ririwpeptide A/B/C  & 4 unknown) | - | 1  (prodigiosin) | - | - | - | 6 | PRJNA554602 |
| ***Serratia marcescens* S7.1** | Mixed salads (fresh vegetables); members of this species can be plant and human pathogens as well as plant growth promoting bacteria | 4 (unknown) | - | 1 (unknown) | - | - | 1 (RiPP-like) | 6 | PRJNA559804 |
| ***Serratia marcescens* 1274** | *Agave sisalana*; members of this species can be plant and human pathogens as well as plant growth promoting bacteria | 4 (unknown) | - | - | 1  (unknown) | - | - | 5 | PRJNA371353 |
| ***Serratia marcescens* 1912768R** | Rhizosphere soil in ginger field; members of this species can be plant and human pathogens as well as plant growth promoting bacteria | 4 (unknown) | - | 1  (prodigiosin) | - | - | - | 5 | PRJNA542556 |
| ***Serratia plymuthica* AS9** | Rape roots and rhizosphere soils of Uppsala (Sweden) | 5 (frederiksenibactin-like & 4 unknown) | - | 3  (zeamine, prodigiosin & unknown) | 1  (unknown) | 1  (sodorifen) | - | 10 | PRJNA60457 |
| ***Serratia plymuthica* A153** | Rhizosphere of wheat  (*Triticum aestivum* L.) | 3 (andrimid, frederiksenibactin-like & unknown) | 1  (oocydin A) | 3  (zeamine & 2 unknown) | - | 2  (sodorifen & unknown) | - | 9 | PRJNA309751 |
| ***Serratia plymuthica* C-1** | Soil collected from a mountain forest | 3  (andrimid, frederiksenibactin-like & unknown) | 1  (oocydin A) | 2  (zeamine & unknown) | 1  (unknown) | - | - | 7 | PRJNA631136 |
| ***Serratia plymuthica* PRI-2c** | Maize rhizosphere soil | 3 (frederiksenibactin-like & 2 unknown) | - | 1 (unknown) | 1  (unknown) | 1  (sodorifen) | 1 (pyrrolnitrin) | 7 | PRJNA84283 |
| ***Serratia plymuthica* S13** | Anthosphere of Styrian oil pumpkin (*Cucurbita pepo* L. subsp. *pepo* var. *styriaca*) | 4 (frederiksenibactin-like & 3 unknown) | - | 2  (zeamine & unknown) | 1  (unknown) | 1  (sodorifen) | - | 8 | PRJNA209109 |
| ***Serratia plymuthica* 3Re4-18** | Endorhiza of *Solanum tuberosum* L. cv. *Cilena* | 4 (enterobactin + 3 unknown) | - | 2  (zeamine & unknown) | 1  (unknown) | 1  (sodorifen) | - | 8 | PRJNA289082 |
| ***Serratia plymuthica* 3Rp8** | Rhizosphere of *Brassica napus* | 4 (frederiksenibactin-like + 3 unknown) | - | 2  (zeamine & unknown) | 1  (unknown) | 1  (sodorifen) | - | 8 | PRJNA289082 |
| ***Serratia plymuthica* 4Rx5** | Soil adhering to oilseed rape's root | 2 (frederiksenibactin-like & unknown) | 1  (oocydin A) | 2 (unknown) | 1  (unknown) | 1  (sodorifen) | 1  (pyrrolnitrin) | 8 | PRJNA417073 |
| ***Serratia plymuthica* 4Rx13** | Rhizosphere of *Brassica napus* | 2 (frederiksenibactin-like & unknown) | 1  (oocydin A) | 2 (unknown) | 1  (unknown) | 1  (sodorifen) | 1  (pyrrolnitrin) | 8 | PRJNA41033 |
| ***Serratia proteamaculans* 336X** | Wheat roots | 3 (minimycin-like, frederiksenibactin-like & unknown) | - | - | 1  (unknown) |  | 1  (pyrrolnitrin) | 5 | PRJNA587215 |
| ***Serratia proteamaculans* 568** | Root endophyte from *Populus trichocarpa* | 5 (minimycin-like, frederiksenibactin-like & 3 unknown) | - | - | 1  (unknown) | - | - | 6 | PRJNA17459 |
| ***Serratia quinivorans* PKL:12** | Rhizospheric soil of *Picrorrhiza kurroa* | 4 (minimycin-like & 3 unknown) | - | - | 1  (unknown) | - | - | 5 | PRJNA529964 |
| ***Serratia rubidaea* NCTC10848** | Cultivated mushroom; members of this species can be human pathogens | 3 (frederiksenibactin-like & 2 unknown) | 2 (unknown) | 1  (prodigiosin) | 1  (unknown) | - | 2  (RiPP-like & pyrrolnitrin) | 9 | PRJEB6403 |
| ***Serratia* sp. AS12** | *Brassica napus* roots | 5 (frederiksenibactin-like & 4 unknown) | - | 3  (zeamine, prodigiosin & unknown) | 1  (unknown) | 1  (sodorifen) | - | 10 | PRJNA60453 |
| ***Serratia* sp. AS13** | Rapeseed plant (*Brassica* *napus*) | 5 (frederiksenibactin-like & 4 unknown) | - | 3  (zeamine, prodigiosin & unknown) | 1  (unknown) | 1  (sodorifen) | - | 10 | PRJNA60455 |
| ***Serratia* sp. FS14** | *Atractylodes* *macrocephala* *Koidz* plant infected by *Fusarium* *oxysporum* | 5 (unknown) | - | 1  (prodigiosin) | - | - | - | 6 | PRJNA198771 |
| ***Serratia* sp. KUDC3025** | Rhizospheric soil of *Artemisia japonica* subsp. *littoricola* | 3 (frederiksenibactin-like & 2 unknown) | 1 (unknown) | 2 (althiomycin & unknown) | (unknown) | - | 2  (RiPP-like & pyrrolnitrin) | 9 | PRJNA555437 |
| ***Serratia* sp. NGAS9** | *Solanum tuberosum* rhizosphere soil | 5 (dichrysobactin,  rhizomide A/ B/C & 3 unknown) | - | 1 (althiomycin) | - | - | - | 6 | PRJNA600387 |
| ***Serratia* sp. S4** | Rhizosphere of naturally growing *Equisetum* plants | 5 (frederiksenibactin-like, minimycin-like & 3 unknown) | - | 1  (unknown) | 1  (unknown) | - | 2  (RiPP-like & pyrrolnitrin) | 9 | PRJNA61833 |
| ***Serratia* sp. 1D1416** | *Euonymus japonicus* gall tissue (mixed culture with *Agrobacterium* *tumefaciens*) | 4 (unknown) | - | - | 1  (unknown) | - | - | 5 | PRJNA493633 |
| ***Serratia surfactantfaciens* YD25** | Rhizosphere soil of Burley tobacco | 4 (rhizomide A/ B/C & 3 unknown) | - | 1  (prodigiosin) | - | - | - | 5 | PRJNA303098 |
| ***Serratia ureilytica* CC119** | Diseased cotton boll; phytopathogen | 5 (rhizomide A/ B/C-like & 4 unknown) | - | 1 (unknown) | - | - | 1  (RiPP-like) | 7 | PRJNA487218 |
| ***Serratia ureilytica* DW2** | *Codonopsis pilosula* rhizosphere soil | 5 (unknown) | - | 1 (unknown) | - | - | - | 6 | PRJNA418349 |
| ***Siccibacter colletis* 1383** | Poppy seeds | 1 (unknown) | - | - | - | 2  (unknown) | 2  (RiPP-like) | 5 | PRJNA246697 |

**^a^**Information extracted from NCBI Biosample and Integrated Microbial Genomes & Microbiomes system (<https://img.jgi.doe.gov/m/>).

^b^In general, we only indicate that it acts as a phytopathogen in cases where there is experimental evidence that a particular isolate causes disease in plants, including its isolation from plants showing disease symptoms. In specific cases, we indicate that members of the species/genus act as food/human/plant-borne pathogens.

**^c^**"Unknown" refers to cases where antiSMASH 7.0 does not associate a biosynthetic gene cluster with a particular metabolite at high confidence level”.
